# SIT 2.0: 21^st^ Century genetic technology for the screwworm sterile-insect program

**DOI:** 10.1186/s12915-016-0310-1

**Published:** 2016-09-19

**Authors:** Luke Alphey

**Affiliations:** The Pirbright Institute, Ash Road, Woking, Surrey GU24 0NF UK

## Abstract

Release of sterile insects, the Sterile Insect Technique (SIT), can be an extremely effective and precise method of pest control. A study in *BMC Biology* from the New World screwworm SIT program and others shows that modern genetic methods can provide major improvements even to this well-established and highly successful SIT program.

See research article: https://bmcbiol.biomedcentral.com/articles/10.1186/s12915-016-0296-8

## Commentary

The Sterile Insect Technique (SIT) is a species-specific, environmentally benign method for controlling insect pests. The method was first developed in the 1950s to control the New World screwworm (*Cochliomyia hominivorax*), the species for which Concha et al. [1] propose an improvement. SIT relies on the release of large numbers of sterile insects to mate with the wild pest population and thereby reduce its reproductive potential. If sufficient wild females mate with sterile males, the target population will decline and collapse. The method has been successfully used to eliminate target populations or, alternatively, to suppress them below an economic threshold, across small and large areas up to continental scale [2].

SIT has several attractive features. For example the control agent—sterile males—will actively seek out wild pest females, including in cryptic or hard-to-reach locations. Furthermore, since these males will mate only with females of their own species, off-target effects on other species are minimized. However, this same feature of species-specificity means that SIT may be most relevant in situations where a single pest species has a large impact.

Despite these benefits, SIT has been used against relatively few insect species, principally the New World screwworm, some tephritids—especially the Mediterranean fruit fly (Medfly *Ceratitis capitata*)—and some moths. Two technical issues have limited uptake of the method; for both, modern genetic methods can provide dramatic improvements [3, 4]. These issues relate to sterilization methods and sex separation. Current large-scale SIT programs still rely on irradiation of pupae or adults to sterilize them before release. Development of the radiation sterilization method was a key breakthrough in the initial development of SIT in the 1950s but it has significant drawbacks. Radiation damages somatic as well as germline cells, so the sterilizing dose of radiation also reduces the performance of the sterilized males, making them less effective. Larger numbers of insects need to be reared and released to compensate for this, making the method more expensive and correspondingly less attractive. Regarding sex separation, male-only releases were shown to be three- to five-fold more effective for the same number of released males in large-scale field trials with Medfly [5]. However, for most pest insects no practical means for large-scale sex separation is available. Sophisticated “genetic sexing” strains were made for this purpose in Medfly [2], but the special chromosomes and mutants required cannot be transferred to other species. Insect synthetic biology offers an alternative and potentially much superior route to the development of such strains and also a means to avoid the need for radiation sterilization. These benefits can be provided by using engineered repressible lethal systems, which kill individuals that carry the synthetic genetic system unless switched off by a repressor, for example, an artificial dietary additive [3, 4]. Such systems can be designed to kill females but not males and/or specific developmental stages, for example.

**Fig. 1.**
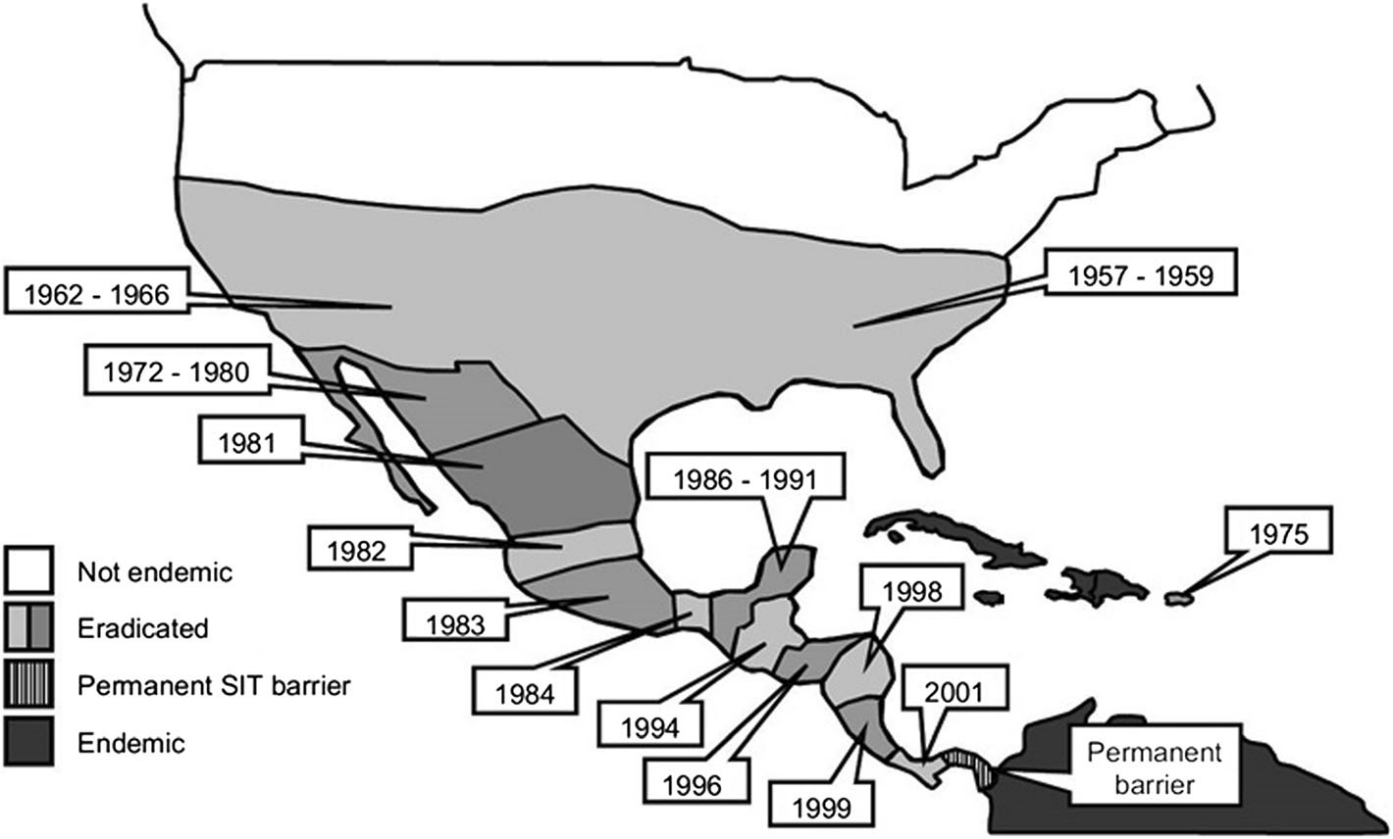
Genetics-based elimination of a major insect pest on a continental scale—the New World screwworm program. Eradication efforts against the New World screwworm started in the southern USA in 1958 after two field trials to assess the effectiveness of the release of sterile insects, then a new concept. The first, on Sanibel Island off Florida in 1953, achieved good sterility but failed to eradicate the pest due to migration from the mainland. A trial in 1954 on the more remote Curacao successfully eliminated the target population—no fertile eggs were detected in sentinel animals after 9 weeks, or three screwworm generations—and the target population was declared eradicated after five months. Following regional successes from 1959, in 1966 the US Department of Agriculture (USDA) was able to declare the entire US free of screwworm. Efforts continued and in 1991 the Mexico-United States Screwworm Commission declared eradication of screwworm from Mexico. The program also provided sterile flies to Libya, where New World screwworm, native only to the Americas, was discovered in 1988—it was eradicated by 1992. Continuing southwards in the Americas, screwworm was eradicated from Belize and Guatemala by 1994, from Honduras and El Salvador by 1996, and so to Panama, where a permanent barrier was established in the early 2000s involving releases of sterile flies by the Panama–US Commission for the Eradication of Screwworm (COPEG) to prevent reinvasion from South America, which remain infested, as do various Caribbean islands (adapted from [11])

Concha et al. [1] describe the application of such methods to the New World screwworm, a major pest of livestock. This is the species for which the SIT was originally developed around 60 years ago. The method was successfully used to eliminate the pest from North and Central America as far south as Panama [6], where a barrier program continues to release sterile males to prevent reinvasion from infested areas to the south (Fig. 1). Concha et al. built a simple genetic circuit comprising a synthetic transcription factor under the control of its own response element. This transcription factor, tTA, has been very widely used in cells and organisms across a wide range of taxa and is generally well tolerated, but this “positive feedback loop” design can drive such high levels of expression as to be lethal. This lethal effect is made female-specific by incorporation of a sex-specific alternative splicing element from a screwworm sex-determination gene so that only females make a mature mRNA that encodes tTA. In the presence of tetracycline, tTA does not bind DNA and therefore tetracycline, provided in the insects’ diet, breaks the positive feedback loop and acts as an antidote to the lethal system. The constructs also contain a marker gene encoding a fluorescent protein so that transgenic individuals are readily distinguished from wild type. The lead strain of Concha et al., FL12-56, carries an insertion which kills females very effectively if present in two copies—if the strain is reared without the antidote, only males survive. This strain can, therefore, provide the major improvements of genetic sexing and a genetic marker but would still need to be radiation sterilized before release. This is because the construct in that strain needs to be present in two copies to be fully effective at killing females in the absence of the chemical antidote that switches off the lethal effect—that’s fine for a production strain, which would be bred so that every individual had two copies—but not in the field as the offspring of released males and wild females will inherit only one copy of the construct.

The molecular designs of Concha et al. are closely based on genetic circuits developed in my laboratory for tephritid fruit flies [7, 8]; though translating this to screwworm is a significant technical accomplishment, perhaps of more interest is that the technical development is closely integrated with the operational Screwworm Eradication Program. The authors developed a panel of candidate strains and put them through a series of performance tests, including tests used by the program for their standard strains. This led to the conclusion that “the results from a battery of fitness tests suggest that some of the transgenic sexing strains could replace the J06 parental wild type strain that is currently used for the SIT program in Panama.” Furthermore, access to program cost data allowed estimates of the potential economic benefits to the program of the incorporation of this genetic technology. The authors conclude that use of their best strain could halve the production costs, a saving of > $20,000 per week based on conservative estimates. While previous laboratory and field trials have demonstrated the effectiveness of this type of genetic approach for suppressing several other major insect pest species, this study provides the first empirically informed evidence of the substantial economic benefits they can provide relative to current SIT practice. Though the actual numbers provided are specific to the screwworm program, the conclusion that even established, successful programs could realise major benefits from incorporating such technology is likely to apply very widely. The strain provides additional benefits beyond cost savings to the program from genetic sexing, such as the presence of a fluorescence marker, which facilitates field monitoring by providing a simple method for distinguishing transgenic and wild insects. Since the engineered strain cannot persist without artificial provision of tetracycline in the larval diet—since this switches off the female-killing gene and is therefore essential for female viability—the hazard posed by insects escaping the facility is greatly reduced relative to the current situation where wild-type insects are reared. The new strain would also greatly increase the effective production capacity of the mass-rearing facility, both by reducing the space needed per million males and through the increased per-male effectiveness of male-only releases. The authors therefore conclude that “the strains developed in this study could make it possible to extend the program to regions where this insect remains a pest.”

Though the strains described are clearly a substantial advance, further improvements are possible. In the lead strain, FL12-56, about one-third of the affected females survive to pupae, the others presumably dying during larval development. If the female-killing effect acted earlier in development, say in embryos or early larvae, the rearing cost would correspondingly be further reduced. More significantly, it should be possible to release such strains, or variants of them, without radiation sterilization. This would avoid the financial costs and negative effects on insect performance of irradiation and therefore provide further cost and effectiveness benefits to the program.

But why bother, one might ask, given the historic success of the program with current technology? Clearly it would be desirable to reduce the current burden of the program on the public purse. Improved technology might also allow expansion to areas that have not yet been feasible, including infested Caribbean islands such as Jamaica and Cuba and areas of South America.

Will these strains, or ones like them, ever make it to the field? The International Atomic Energy Agency (IAEA) together with the US Dept of Agriculture (USDA), the major technology developer for SIT, have had potentially useful transgenic strains of relevant species for well over 10 years without visible progress to field use. However, several factors suggest that this case may be different. The USDA program may not be wedded to a particular technology as the IAEA is to radiation. Several field trials have been successfully completed with other engineered insects, thereby providing technical and regulatory precedents. The first of these trials involved the USDA pink bollworm SIT program [9]; the strain carried a genetic marker useful for distinguishing released from wild males but needed to be radiation sterilized. In subsequent trials with an engineered strain of the mosquito *Aedes aegypti* carrying a conditional lethal construct, OX513A, we showed that this could replace radiation sterilization [10] and provide superior control of this key vector of yellow fever, dengue, and Zika viruses relative to current methods. Concha et al. [1] report that an application for conducting open field trials with radiation sterilized transgenic male screwworm flies has been submitted to the Panamanian Government.

Long before any of these strains were made it was proposed that several major benefits could be provided to classic SIT by the incorporation of modern genetic methods [3, 4]. These included (i) the provision of a genetic marker to facilitate discrimination of released sterile insects from wild fertile ones; (ii) removing the need for radiation sterilization and thereby increasing the range of pest species to which the method could effectively be applied; and (iii) efficient “genetic sexing” to allow male-only releases of sterile insects. The first two features have already been demonstrated in the field; the proposed trials with these screwworms would address the third. The relative ease with which these constructs and designs can be transferred between species greatly facilitates the application of sterile male methods to pest species not currently controlled by such means. Where SIT is already in use, Concha et al. show that the economic case for uptake of such methods is compelling, at least for the grandfather and flagship of them all, the New World screwworm program.
